# Adaptive Density Spatial Clustering Method Fusing Chameleon Swarm Algorithm

**DOI:** 10.3390/e25050782

**Published:** 2023-05-11

**Authors:** Wei Zhou, Limin Wang, Xuming Han, Yizhang Wang, Yufei Zhang, Zhiyao Jia

**Affiliations:** 1 School of Computer Science and Technology, Changchun University of Science and Technology, Changchun 130022, China; 2020200125@mails.cust.edu.cn (W.Z.);; 2 School of Information, Guangdong University of Finance & Economics, Guangzhou 510320, China; 3 College of Information Science and Technology, Jinan University, Guangzhou 510632, China; 4 College of Information Engineering, Yangzhou University, Yangzhou 225127, China; 5 School of Economics and Management, Shenyang University of Chemical Technology, Shenyang 110142, China; z2021585@stu.syuct.edu.cn

**Keywords:** adaptive clustering, DBSCAN, chameleon swarm algorithm, parameter optimization, image segmentation

## Abstract

The density-based spatial clustering of application with noise (DBSCAN) algorithm is able to cluster arbitrarily structured datasets. However, the clustering result of this algorithm is exceptionally sensitive to the neighborhood radius (*Eps*) and noise points, and it is hard to obtain the best result quickly and accurately with it. To solve the above problems, we propose an adaptive DBSCAN method based on the chameleon swarm algorithm (CSA-DBSCAN). First, we take the clustering evaluation index of the DBSCNA algorithm as the objective function and use the chameleon swarm algorithm (CSA) to iteratively optimize the evaluation index value of the DBSCAN algorithm to obtain the best *Eps* value and clustering result. Then, we introduce the theory of deviation in the data point spatial distance of the nearest neighbor search mechanism to assign the identified noise points, which solves the problem of over-identification of the algorithm noise points. Finally, we construct color image superpixel information to improve the CSA-DBSCAN algorithm’s performance regarding image segmentation. The simulation results of synthetic datasets, real-world datasets, and color images show that the CSA-DBSCAN algorithm can quickly find accurate clustering results and segment color images effectively. The CSA-DBSCAN algorithm has certain clustering effectiveness and practicality.

## 1. Introduction

With the continuous development of society and rapid changes in Internet technology, big data has come into people’s view and developed rapidly, which has a very broad prospect for social progress [1]. Data mining techniques can extract various correlations and validity information from massive data to enhance the value of the data [2]. Clustering is one of the key techniques in data mining [3]. Clustering algorithms can categorize data with unknown relationships based on the similarity between the data and then mine the valuable information hidden in the data. Clustering algorithms have promising applications in the fields of face recognition [4], image segmentation [5], stock prediction [6], and medical image processing [7].

Clustering algorithms can generally be used in partition-based [8], hierarchy-based [9], density-based [10], grid-based [11], model-based [12], and graph-based [13] ways to perform classification. The k-means algorithm is the most traditional method of partitioning clusters [14]. Affinity propagation (AP) clustering is a method based on the transfer of information between data points [15]. DBSCAN is a classical density-based clustering method that can identify arbitrarily shaped classes with noisy points in spatial data [16]. The DBSCAN method seeks to uncover potentially meaningful messages in a given dataset. This method can effectively handle anomalous data by connecting neighboring regions with high densities in a given spatial database. In addition, the DBSCAN clustering algorithm has the benefits of higher efficiency and wider applicability as it can effectively handle noisy points without inputting the number of clustering centers.

Swarm intelligence is a computational method that simulates the smart behavior of biological groups; this method has the advantages of fast convergence, high solution accuracy, and robustness and is suitable for solving large-scale complicated problems and optimization issues. Typical swarm intelligence algorithms include particle swarm optimization [17], the cuckoo search algorithm [18], and the bird swarm algorithm [19]. In recent years, researchers have proposed a chameleon swarm algorithm (CSA) based on the feeding behavior of chameleons under different conditions [20].

The DBSCAN algorithm parameter *Eps* is exceptionally sensitive to clustering results and is prone to the problem of the over-identification of noise points during the clustering process. To solve the above problems, we use the CSA method to perform an adaptive search for the clustering parameter *Eps* of DBSCAN and obtain the best clustering parameter value by continuous iterative optimization. The initial clustering results can be obtained by inputting the optimized parameters into DBSCAN. To prevent the algorithm from over-identifying boundary points as anomalies, we use the nearest neighbor mechanism to assign the noise points of the optimization algorithm to the nearest category points, forming the final CSA-DBSCAN algorithm.

Although the DBSCAN method has a very good clustering effect, it still has some drawbacks. To address the challenges DBSCAN faces when handling large natural datasets that are fuzzy, Smiti et al. [21] proposed the “Soft DBSCAN” method using the fuzzy membership function of fuzzy c-mean theory. This method can cluster arbitrary shape data and has a better processing effect on noise points. Furthermore, DBSCAN performs neighborhood queries for every object in the dataset, resulting in low efficiency; to address this problem, Severino et al. [22] proposed a most-related region search strategy for the DBSCAN method. The approach checks the neighborhoods of a subset of objects in the dataset to improve the clustering efficiency. All of the above algorithms significantly improve the performance of the DBSCAN algorithm but do not address one key problem, which is that the parameters of the DBSCAN algorithm are exceptionally sensitive to the clustering results. Compared with the above literature, the CSA-DBSCAN algorithm can use the CSA algorithm with its excellent global search capability to perform an adaptive search on the *Eps* parameter in the DBSCAN method and can accurately find the optimal position without setting a specific search range.

The parameter problem of the DBSCAN algorithm has always been a hot topic for scholars at home and abroad. Bryant et al. [23] proposed a heuristic approach for RNN-DBSCAN. The algorithm utilizes the core observe and observe achievability definitions based on inverse nearest neighbors, which reduces the complexity of the problem (i.e., compared to the two parameters *Eps* and *MinPts*, RNN-DBSCAN needs to use only one parameter *k*). Hou et al. [24] proposed a parameter-free clustering method for DSets-DBSCAN. The algorithm first equalizes the histogram of pairwise similarity matrices used for the input data to obtain the dominant set (*dset*) and finally uses the *dset* to automatically determine the parameters to extend the clusters. All the above studies reduced the influence of parameters on clustering results. However, the best values of clustering parameters could not be found quickly and accurately. Compared with the above literature, the use of the CSA method to deal with the parameter problem of DBSCAN can greatly reduce the time consumption of the tuning process.

Since the swarm intelligence algorithm has a good global search capability, many scholars use this method to adaptively search for the parameters of the density clustering algorithm [25,26,27,28]. Zhu et al. [29] proposed the K-DBSCAN algorithm. The algorithm achieves a better clustering property by applying the proposed new harmony search method to DBSCAN to optimize the parameters “*Eps*” and “*MinPts*” and by using a multi-objective co-optimization function. Wang et al. [30] proposed a parameter-adaptive DBSCAN algorithm for bird swarm optimization. The algorithm finds the exact resulting clusters corresponding to the optimal *Eps* parameters of the DBSCAN algorithm by continuously adjusting the search range. All the above algorithms solve the parameter setting problem in different ways, but they all lack the mechanism to deal with the identified anomalies. The CSA-DBSCAN algorithm uses the nearest neighbor mechanism and is able to solve the problem of the over-identification of noise points and the inability to obtain an effective assignment of anomalies.

To further measure the compactness relationship between noise and boundary points, we introduce the theory of deviation of spatial data in the nearest neighbor mechanism. Finally, an advanced adaptive CSA-DBSCAN method is developed and applied to color image segmentation. We test the CSA-DBSCAN algorithm on synthetic datasets and real-world datasets and find that the improved method has better clustering accuracy and a better clustering effect than other algorithms for data with arbitrarily complex structures. To improve the computational efficiency of DBSCAN technology in image processing, we use the simple linear iterative clustering (SLIC) method to construct superpixel information [31] and then used an improved method to segment the superpixel data according to the characteristics of the image color space to realize image segmentation by the adaptive CSA-DBSCAN method. The practicality and effectiveness of the improved method are confirmed by the comprehensive evaluation of the clustering effect and image segmentation performance. Through comparison and analysis with other advanced clustering algorithms, the superior performance of the improved algorithm in adaptive optimization and image segmentation is verified. The main contributions to this article are presented as follows:

1. The CSA-DBSCAN method is proposed to realize the parameter adaption of the clustering process and reduce the parameter tuning complexity.
2. The nearest neighbor mechanism is used to assign the noise points identified by the CSA-DBSCAN method to the nearby clusters, which addresses the issue of assigning the noise points of the DBSCAN algorithm.
3. Deviation theory is introduced in the nearest neighbor mechanism to further measure the compactness between data points.
4. The construction of color image superpixel information using the SLIC algorithm increases the clustering performance of the CSA-DBSCAN algorithm in color image segmentation.

The rest of this article is organized as follows. Section 2 explain the theory of DBSCAN and the chameleon swarm method (CSA) in depth. Section 3 analyzes the newly presented adaptive DBSCAN method based on the CSA method (CSA-DBSCAN). Section 4 experiments with and summarizes the performance of CSA-DBSCAN in various datasets and evaluates it against other methods. Finally, conclusions and future progress are given in Section 5.

## 2. Research Background

In this section, we give two basic approaches related to our proposed algorithm. The first is the DBSCAN method, and the second is the chameleon swarm optimization algorithm.

### 2.1. Density-Based Spatial Clustering of Application with Noise

Esterl et al. [16] proposed a popular and highly efficient DBSCAN method. The method can handle sample sets of any shape and size, and the clustering results are independent of the input order of data objects, with good interference resistance. The core principle of the method is that for each target cluster, there are a series of sets containing a specified point threshold within a given neighborhood radius. The clustering efficacy of the method depends entirely on two parameters: the minimum number of objects in the search radius (*MinPts*) and *Eps*. The number of *MinPts* generally takes the value of 4 and is not very sensitive to the results of the process, which facilitates the parameterization of the method. The parameter *Eps* is very sensitive to the clustering performance, which causes some difficulty when setting the *Eps* parameter.

Depending on the aforementioned two parameters, we can classify the points in the sample into three types.

- Core point: If the quantity of sample points in the *Eps* neighborhood is greater than *MinPts*, the points of this type are called core points.
- Border point: The points within the radius *Eps* are smaller than *MinPts*. The boundary and some core points within the cluster are mutually contained within the *Eps*.
- Outlier: A single site that does not satisfy the core point condition or the bounding point condition.

Since the DBSCAN method marks three types of points by progressively searching for them, there are three descriptions of the point-to-point density relationship in the search process. Suppose that given a dataset $S=\{y_{1},y_{2},\cdots ,y_{n}\}$, the relationship between data points is described as follows:

- Direct density reachable: If $y_{j}$ is within the range of the *Eps* of $y_{i}$, and $y_{i}$ is a core point, $y_{j}$ is said to be reachable by the direct density of $y_{i}$.
- Density reachable: For points $y_{i}$ and $y_{j}$, if there exists a sequence $p_{1},p_{2},\cdots ,p_{n}$, where $p_{1}=y_{i},p_{n}=y_{j}$, and $p_{n+1}$ is directly density reachable by $p_{i}$, we say $y_{i}$ is density reachable by $y_{j}$.
- Density connected: For $y_{i}$ and $y_{j}$, $y_{i}$ and $y_{j}$ are called connected by density if the existence of $y_{k}$ makes both $y_{i}$ and $y_{j}$ realizable by the density $y_{k}$.

### 2.2. Chameleon Swarm Algorithm

In 2021, inspired by the foraging behavior of chameleons, Braik [20] proposed a novel chameleon swarm optimization algorithm (CSA). This method focuses on modeling the dynamic behavior of chameleons as they search for food near forests, dunes, and marshes. This method of math modeling mimics the process of chameleons searching for food, in which they rotate their eyes nearly 360 degrees for prey localization and capture prey with their sticky tongue that fires at high speed.

In nature, chameleons live in a variety of places, such as lowlands, rainforests, deserts, semi-deserts, and even mountains. Chameleons are very good climbers and have excellent eyesight, being able to see up to 32 feet in front of them. Chameleons usually feast upon insects such as sulfur worms, praying beetles, grasshoppers, and crickets. Some big individuals even use lizards and baby birds to improve their diet. The individual first uses their eyes to locate their target and then uses their tongue to capture it to eat and seek optimal for the solving way. On the flip side, chameleons have some natural predators, such as snakes, vultures, and lemurs. Chameleons can adapt their color to their surroundings, which is very beneficial when hunting or escaping from natural predators. The CSA method is particularly unique in its advantages based on the unique capabilities of the chameleon.

- Initialization: This method, as with other optimization algorithms, is initialized randomly in the search range.
  (1) $$y_{t}^{i}=\left[y_{t,1}^{i},y_{t,2}^{i},\cdots ,y_{t,d}^{i}\right]$$
  (2) $$y^{i}=l_{j}+\left(u_{j}-l_{j}\right)\times r$$
  where *t* is the numeration of iterations, i={1,2,⋯,n}, *d* denotes the dimension, and $y_{t,d}^{i}$ is the location of the individual *i* in dimension *d*. In Equation (2),  *r* denotes the creation of random numbers in the range [0,1], $y^{i}$ denotes the initial level of the *i*th individual, and $u_{j}$ and $l_{j}$ are the upper and lower bounds of dimension *j*, respectively.
- Search for prey: At this point, the chameleon begins to search for prey in order to solve the problem of needing food.
  (3) $$y_{i,j}^{t+1}=\left\{\begin{array}{cc} y_{i,j}^{t}+r_{2}\left(P_{i,j}^{t}-G_{i}^{t}\right)p_{1}+r_{1}\left(G_{j}^{t}-y_{i,j}^{t}\right)p_{2} & r_{i}\geq P_{p}\\ y_{i,j}^{t}+\mu \left(l_{b}^{j}+r_{3}\left(u^{i}-l^{i}\right)\right)sgn\left(rand-0.5\right) & r_{i}<P_{p} \end{array}\right.$$
  where $y_{i,j}^{t+1}$ donates the position of chameleon *i* at step (t+1) in dimension *j*, $G_{j}^{t}$ is the current optimal individual position, $P_{i,j}^{t}$ is the best position of chameleon *i* so far, $p_{1}$ and $p_{2}$ are two parameters controlling the exploration ability, $r_{1}$, $r_{2}$, and $r_{3}$ are all random values within [0,1], and $r_{i}$ looks different from the first three but is also a random quantity within [0,1]. $P_{p}=0.1$ indicates the probability that the chameleon perceives its prey. sgn(rand−0.5) is 1 or −1, which mainly affects the direction of exploration and development. μ is calculated as follows:
  (4) $$\mu =\gamma e^{{\left(-\alpha t/T\right)}^{\beta }}$$
  where γ, α, and β are 1, 3.5, and 3, respectively, *t* is the current number of iterations, and *T* is the maximum number of iterations.
- Prey location: The eyes of chameleons move independently, which allows them to skillfully explore spatially and find prey. These eyes can view in two different directions and rotate and focus simultaneously, allowing them to see a sweeping view of their environments.

To simulate this process, the authors performed four setups: translating the location of the original chameleon to its gravity center, locating the matrix of rotations that recognizes the location of the prey, updating the chameleon’s position using the matrix of rotations at the gravity center, and moving the chameleon back to its original position.

After the above process, we started the step that involved updating the chameleon’s position. This stage simulates the position update that occurs when the chameleon locates its prey by eye rotation.

(5) $$y_{t+1}^{i}=yr_{t}^{i}+{\bar{y}}_{t}^{i}$$

where ${\bar{y}}_{t}^{i}$ is the average location of the individual in each dimension before position rotation, $y_{t+1}^{i}$ is the coordinates after rotation, and $yr_{t}^{i}$ is the coordinates of the center of rotation, specified as follows:

(6) $$yr_{t}^{i}=m\times yc_{t}^{i},$$

where $yr_{t}^{i}$ is the centering coordinate.

(7) $$yc_{t}^{i}=y_{t}^{i}-{\bar{y}}_{t}^{i}$$

(8) $$m=R(\theta ,\vec{V}z_{1},{\vec{z}}_{2})$$

where *m* denotes the rotation matrix of the chameleon position rotation, $\vec{V}z_{1}$ and ${\vec{z}}_{2}$ represent two quadrature variants in the spatial coordinates, and *R* denotes the rotation matrix on each axis.

(9) $$\theta =rsgn\left(rand-0.5\right)\times 180^{\circ }$$

where θ represents the rotation angle of the chameleon’s eyes, and *r* is a random value within [0,1], so that θ can be limited to $\left[-180^{\circ },180^{\circ }\right]$.

- Hunting prey: The algorithm determines that the chameleon close to the prey is the best-positioned chameleon. This chameleon attacks its prey with its tongue. Therefore, its position will be slightly updated, since its tongue can extend to twice its original length. The speed of the chameleon’s tongue as it moves toward its prey is defined as:

(10) $$v_{t+1}^{i,j}=\omega v_{t}^{i,j}+r_{1}\left(G_{t}^{j}-y_{t}^{i,j}\right)c_{1}+r_{2}\left(P_{t}^{j}-y_{t}^{i,j}\right)c_{2}$$

Many of these parameters were previously described. $c_{1}=c_{2}=1.75$ controls the effect of *G* and *P* on the velocity of the tongue. ω is updated as follows:

(11) $$\omega ={\left(1-t/T\right)}^{\left(\rho \sqrt{\left(t/T\right)}\right)}$$

where ρ=1 is the parameter that controls the exploitation capacity. When the chameleon’s tongue is projected towards the prey, the acceleration of the tongue bouncing up is *a*. The position of the tongue indicates the position of the chameleon, and the formula is as follows:

(12) $$v_{t+1}^{i,j}=y_{t}^{i,j}+\left({\left(v_{t}^{i,j}\right)}^{2}-{\left(v_{t-1}^{i,j}\right)}^{2}\right)/\left(2a\right)$$

(13) $$a=2590\times (1-e^{-log(t)})$$

At this point, the CSA algorithm has completed the merit-seeking foraging mechanism of the chameleon. Next, we will further illustrate the algorithm fusion process of CSA-optimized DBSCAN.

## 3. Materials and Methods

In this section, we show the technical details of the present DBSCAN method based on chameleon optimization (CSA-DBSCAN). Firstly, we focus on the theoretical model and optimization details of the adaptive parameter search system. Then, we obtain the best processing results corresponding to the best parameter values. To further evaluate the potential relationship between the noise and boundary points, we use the nearest neighbor approach to assign the noise points to the nearest boundary points. Finally, we introduce the theory of deviation in the process of measuring data similarity to evaluate the compactness between data, which provides a more reliable reference basis for the assignment of noise points.

### 3.1. Adaptive Parameter Seeking Strategy

DBSCAN is an elegant method that allows the clustering of data with arbitrary shapes and constructions. However, when testing the superiority of the clustering capability of a dataset, two parameters need to be jointly adopted to achieve better clustering results. This adds, to some extent, to the complexity and time consumption of clustering tuning parameters. In the clustering process, we usually choose the value of *MinPts* as a positive integer from 1 to 15. However, the value of *Eps* is exceptionally sensitive to the clustering outcomes. When using a dataset, the value of *Eps* might be taken to be as precise as 0.01. This poses a challenge and difficulty in the selection of parameters for the DBSCAN method.

To overcome this limitation, we used the chameleon optimization algorithm with its excellent search power to solve the parameter-setting task by finding the best arguments for every dataset. To this end, we proposed an adaptability mechanism for density clustering parameters based on chameleon optimization. First, we set the value of the argument *Eps* as the position of the initial chameleon population. We first initialized a certain amount of *Eps* populations as candidate resolutions to further search for the location of the optimality parameter. Since there is only one parameter to be searched for, we set the dimension as 1. We set the size of the population as *n* and the current quantity of iterations as *t*. Then, the candidate solutions for the *Eps* parameter in the one-dimensional space can be expressed by Equation (14).

(14) $$E_{t}^{i}=\left[E_{t,1}^{1},E_{t,1}^{2},\cdots ,E_{t,1}^{n}\right]$$

In Equation (14), *E* denotes the candidacy solution of the *n Eps* of the chameleon groups. To facilitate the search for the best parameter, we set the cluster evaluation index value as the fitness value to be optimized by the CSA method. The chameleon optimization technique has a very sharp global search performance, which can balance the accuracy of the search procedure by adjusting the position of the most optimal individual in all directions. After running the continuous iterative process of the method and updating the size and position of the fitness value, we very quickly obtained the optimal population position corresponding to the *Eps* parameter and thus obtained the optimized clustering effect.

In data mining clustering tasks, different clustering evaluation metrics are available to further measure the ability of an algorithm to deal with a given problem. To facilitate testing the clustering performance of the proposed method, we used the obtained adjusted Rand index (ARI) as an adaptation function to verify the clustering performance. In addition, to test the clustering effect of the CSA-DBSCAN method in image segmentation, we used the most popular boundary displacement error (BDE) [32] as the fitness function to measure the method’s effectiveness in image segmentation. Therefore, we set a total of two fitness functions that solve the different problems of the algorithm.

Suppose *a* denotes the number of identical samples in the real label *A* and the clustered label *B*, and *b* denotes the number of different samples in *A* and *B* and the total number of *n*. To observe the clustering performance more carefully, the external evaluation index ARI can be used as the fitness function. ARI is a range of values (0,1), and the closer the value is to 1, the better the clustering effect. Therefore, in this subsection, we reset the ARI index fitness function, which is set as follows:

(15) $$Fit_{ARI}=\frac{max(RI)-E[RI]-(RI-E[RI])}{max(RI)-E[RI]}\times 100\%$$

(16) $$RI=\frac{a+b}{C_{n}^{2}}$$

To verify the effectiveness of the CSA-DBSCAN algorithm in practical applications, we used real color images to verify the ability of the CSA-DBSCAN method to perform image segmentation. The fitness function for image segmentation is set as follows.

(17) $$Fit_{BDE}=\frac{\sum_{i}^{N_{2}}d\left(p_{i},B_{1}\right)/N_{2}+\sum_{i}^{N_{1}}d\left(p_{i},B_{2}\right)/N_{1}}{2}$$

(18) $$d\left(p_{i},B_{2}\right)=min_{p\in B_{2}}∥p_{i}-p∥$$

where *d* is the length from the boundary point $p_{i}$ in $B_{1}$ to the boundary set $B_{2}$, and $N_{1}$ and $N_{2}$ are the number of boundary sets $B_{1}$ and $B_{2}$.

The BDE is usually greater than 1; the lower the metric value, the better the image segmentation effect. Therefore, this section directly uses the BDE as the fitness function for the adaptive search.

The CSA-DBSCAN algorithm allows different fitness functions to be used according to their actual application environment. Different fitness functions can solve the issue of parameter setting for the DBSCAN method in different problems to avoid the influence of artificial parameter settings on experimental results. Therefore, this method improves the precision of clustering and the clustering effect. This adaptive CSA-DBSCAN method also has a good clustering effect in color image segmentation.

This subsection achieved the cross-fertilization of disciplines by fusing swarm intelligence theory and clustering methods and solved the problem related to the adaptive selection of the clustering parameter. The final parameter-seeking system based on chameleon optimization was thus obtained.

### 3.2. Noise Point Allocation Mechanism

The nearest neighbor mechanism is an extension of the *k*-nearest neighbor (KNN) method [33], which is a prominent and simple intelligent classification approach. It is a theoretically mature method. It is both one of the easiest machine learning methods and the most basic of the instance-based teaching methods, as well as one of the finest text categorization methods.

The underlying principle is that if the *k* most resembling examples of an instance in the spatial characteristic belong to a certain class, then that example also falls into that class. The chosen neighbors are all instances that have been properly categorized. This method supposes that all examples respond to points in the *N*-dimensional Euclidean spatial aspect. By calculating the separation between one point and every other point, the *k* nearest sites to that point are taken out, and then the point that belongs to the largest proportion of the categories within these *k* points is counted; that point then belongs to that category.

While obtaining the optimal resulting clusters for the best parameter values, we also saw the noise points identified by the method. To further explore the hidden relationship between noise points and boundary points, we used the nearest neighbor mechanism to assign noise points to the nearest clusters. The nearest neighbor mechanism evolved from the k-nearest neighbor method. The result of DBSCAN after an adaptive search has fewer noisy points, and the distance to the nearest boundary point is much smaller than the distance to other points. In this work, we unified the assignment of anomalies to the nearest boundary points based on the nearest neighbor feature to better measure the potential relationship between noise points and boundary points.

First, we calculated the separation matrix among the data sites and then sorted the separation matrix from smallest to largest. Then, we extracted the size and the original position of the distance matrix separately for storage. To obtain the data characteristics of the noise points, we extracted all the positions corresponding to the distance matrix with category points, and finally, we obtained the distance matrix and the corresponding positions of the noise points. We based our judgment on the first column of the noise point position (i.e., the point closest to the boundary). Assigning it to the nearest category completed the assignment of noise points.

In the noise point assignment strategy, the distance was calculated in such a way that we usually used the Euclidean distance. Suppose there is a dataset $S=\{x_{1},\cdots ,x_{i},\cdots ,x_{N}\}$, and *N* is the number of data points. Then, in *d* dimensions, $x_{i}$ and $x_{j}$ are $x_{i}=\left\{x_{i1},x_{i2},\cdots ,x_{id}\right\}$ and $x_{j}=\left\{x_{j1},x_{j2},\cdots ,x_{jd}\right\}$, respectively. The Euclidean distance is as follows:

(19) $$d_{ij}=\sqrt{\sum_{k=1}^{d}{\left(x_{ik}-x_{jk}\right)}^{2}}$$

In Equation (19), $d_{ij}$ denotes the distance between different data points. This calculation only measures the distance between data points and does not describe the compactness of the relationship between data points. To solve this problem, we introduced deviation theory in the nearest neighbor assignment mechanism and used this theory to measure the compactness between data points in an old way. The deviation theory is calculated as shown below.

(20) $$de_{i}=\frac{d_{ij}-mean\left(d_{ij}\right)}{N}$$

where $mean(d_{ij})$ denotes the average of the data point distances, *N* represents the overall quantity of data points, $d_{ij}-mean\left(d_{ij}\right)$ represents the degree of deviation of the data points from the average, and $de_{i}$ denotes the value of the attribute used to describe the compactness of the current data point obtained after obtaining the distance between data point *i* and the other data points.

Equation (20) can better help the nearest neighbor assignment of anomalies. Using $de_{i}$ instead of the Euclidean distance can further measure the compactness between anomalies and boundary points, which helps in anomaly assignment. Finally, using adaptive parameter optimization, anomaly assignment strategy, and deviation theory, the CSA-DBSCAN method was proposed.

**Figure 1:**
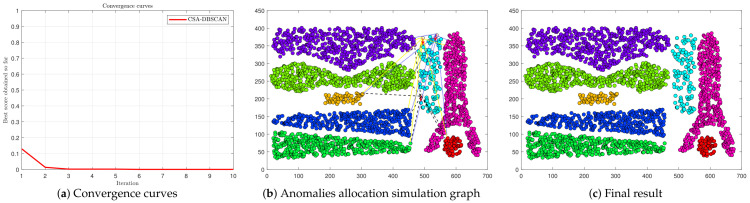
The anomaly assignment process on the Complex8 dataset.

In Figure 1, we give the anomaly assignment process for the Complex8 dataset. We give the convergence curve of the CSA-DBSCAN algorithm (see Figure 1a). In Figure 1a, the CSA-DBSCAN algorithm searches for the optimal parameter positions when the number of iterations is 3. To facilitate understanding, we give a simulation graph of the anomaly assignment process of the CSA-DBSCAN algorithm. In Figure 1b, the three points in white indicate the anomalies identified by the DBSCAN algorithm after iteration to obtain the best parameters. The red arrows indicate that the anomalies are assigned to the nearest category points after the compactness degree calculation of the fusion deviation theory. The yellow solid line, black dashed line, and blue dotted line indicate the degree of compactness (also called nearest deviation distance) between the anomalies and other category points, respectively. The CSA-DBSCAN algorithm assigned the anomalies to the category points with the nearest deviation distance by comparing them with the deviation distance of other data points from different categories. The final clustering results were obtained by assigning the anomalies (see Figure 1c).

In Algorithm 1, we give the specific flow of the CSA-DBSCAN method. From the algorithm flow, we know that the time complexity of updating the position of the CSA-DBSCAN algorithm population when searching for prey with the number of iterations *I* is O(In), the time complexity of updating the position of eye rotation is O(In), and the time complexity of updating the speed and position of the tongue attack on prey is O(In). Since the time complexity of the DBSCAN algorithm is O(nlogn), the time complexity of the CSA-DBSCAN algorithm can be calculated as O(In)+O(In)+O(In)+O(Inlogn) ≈ O(Inlogn).
**Algorithm 1** The proposed CSA-DBSCAN method.**Input:** Dataset *S*, density number MinPts, problem dimension *d*, maximum iteration number *I*, chameleons number *n*, search bounds *u* and *l*, position update probability $P_{p}$, and random numbers $r_{1}$, $r_{2}$, $r_{3}$, and $r_{i}$**Output:** The optimal *Eps*, best cluster result 1:The DBSCAN evaluation index is used as the object function to evaluate the adaptation value 2:Use the value of *Eps* as the location of the chameleon 3:**while** t<I **do** 4:    Using Equations (4), (11), and (13) to define parameters μ, ω, and *a* 5:    **for** i=1:n **do** 6:        **for** j=1:d **do** 7:           **if** $r_{i}\geq P_{p}$ **then** 8:               According to Equation (3), $y_{i,j}^{t+1}=y_{i,j}^{t}+r_{2}\left(P_{i,j}^{t}-G_{i}^{t}\right)p_{1}+r_{1}\left(G_{j}^{t}-y_{i,j}^{t}\right)p_{2}$; 9:           **else**10:               According to Equation (3), $y_{i,j}^{t+1}=y_{i,j}^{t}+\mu \left(l_{b}^{j}+r_{3}\left(u^{i}-l^{i}\right)\right)sgn\left(rand-0.5\right)$;11:           **end if**12:        **end for**13:    **end for**14:    **for** i=1:n **do**15:        $y_{t+1}^{i}=yr_{t}^{i}+{\bar{y}}_{t}^{i}$16:    **end for**17:    Determine the speed of the chameleon when attacking the prey at the optimal *Eps* position according to Equation (10)18:    Determine the optimal chameleon location according to Equation (12)19:    Update the new *Eps* position in the CSA-DBSCAN algorithm20:    t=t+121:**end while**22:Assign noise points using the nearest neighbor mechanism and deviation theory23:Obtain the best clustering result

## 4. Results and Discussion

In this paper, we tested the clustering performance of the improved method using synthetic and real-world datasets and applied it to a clustering task on the Berkeley image segmentation dataset (BSDS500) [34] to further validate the utility of the CSA-DBSCAN algorithm. We demonstrated the effectiveness of the new adaptive method for outlier assignment on datasets with more complex density structures. We compared the clustering results of the proposed algorithm with the classical k-means [14], AP [15], DBSCAN [16], DPC [10], DPCSA [35], AmDPC [36], and DeDPC [37] algorithms for analysis. Among them, DPCSA, AmDPC and DeDPC are the most advanced clustering algorithms proposed in recent years. In this experiment, we compared and analyzed the values of the normalized mutual information (NMI), adjusted Rand index (ARI), and F-measure (FM) evaluation metrics of the above four mentioned algorithms, respectively, to further evaluate the clustering ability of these different algorithms. As shown in Table 1, we used twelve datasets to measure the efficiency of the method. As also shown in Table 1, the former six were synthetic datasets, and the latter six were real-world datasets. All datasets are described in detail below.

### 4.1. Evaluation of Clustering Effectiveness

The experimental environment was simulated using a computer with an Intel Pentium 2.9 GHz, 8.00 GB memory, and 500 GB hard drive running a Windows 10 operating system and using the MATLAB2019a programming language.

A detailed description of the clustering performance evaluation metrics is given before we derive the experimental results. We employed the three most valid cluster performance metrics (NMI, ARI, ACC, and FM). These metrics take values between −1 and 1, with values nearer to 1 indicating the better performance of the clusters.

NMI is a very valuable external evaluation metric of clustering. Suppose the true label of a dataset is *A*, and the clustering result label is *B*. Then, the unique values in *A* are extracted to form vector *E*, and the unique values in *B* are extracted to form vector *F*. The NMI of *A* and *B* are as follows:

(21) $$NMI\left(A,B\right)=\frac{I(E,F)}{\sqrt{H(E)H(F)}}$$

(22) $$I\left(E,F\right)=\sum_{e\in E}\sum_{f\in F}log\left(\frac{p(e,f)}{p(e)p(f)}\right)$$

(23) $$H\left(E\right)=-\sum_{1}^{n}p\left(e_{i}\right)\log_{2}p\left(e_{i}\right)$$

(24) $$H\left(F\right)=-\sum_{1}^{n}p\left(f_{i}\right)\log_{2}p\left(f_{i}\right)$$

where p(e) indicates the probability for *e* in *A*, and p(f) denotes the proportion of *f* in *B*. P(e,f) indicates the joint probability of *e* and *f*.

The Rand index (RI) uses a paired approach to detect true negatives (TN), true positives (TP), false negatives (FN) and false positives (FP). In clustering methods, the metric values of the RI tend to have high values, and it is difficult to distinguish the effectiveness of the methods. Therefore, we used the ARI to calculate the algorithm performance instead of RI.

(25) $$RI=\frac{TP+TN}{TP+TN+FP+FN}$$

(26) $$ARI=\frac{RI-E[RI]}{max(RI)-E[RI]}$$

ACC is a classical evaluation index often used to measure clustering accuracy. Assuming that $r_{i}$ and $s_{i}$ represent cluster labels and real labels, respectively, in a dataset of *n* samples, ACC can be defined as:

(27) $$ACC=\frac{\sum_{i=1}^{n}\delta \left(s_{i},map\left(r_{i}\right)\right)}{n}$$

(28) $$\delta \left(x,y\right)=\left\{\begin{array}{cc} 1 & if\;x=y\\ 0 & otherwise \end{array}\right.$$

where $map(r_{i})$ is a permutation mapping function that matches the cluster label and the real label.

Assume that the real label of the dataset is *A* and the label of the clustering is *B*. $a_{1}$ denotes the quantity of samples that belong to the same clusters in both *A* and *B*. $a_{2}$ indicates the quantity of samples that belong to the same clusters in *A* and not in *B*. $a_{3}$ indicates the quantity of samples that belong to different clusters in *A* but belong to the same clusters in *B*. FM was calculated as shown below.

(29) $$FM=\sqrt{\frac{a_{1}}{a_{1}+a_{2}}\frac{a_{1}}{a_{2}+a_{3}}}$$

### 4.2. Performance Evaluation on Synthetic Datasets

We validated the clustering performance of the CSA-DBSCAN algorithm on synthetic datasets. The external evaluation index (ARI) was chosen as the fitness function ($Fit_{ARI}$) for this experiment. We selected Donutcurves, Target, Pearl, Complex8, and Complex9 datasets, all of which have complex density structures, to observe the ability of the CSA-DBSCAN method to handle complex data.

**Figure 2:**
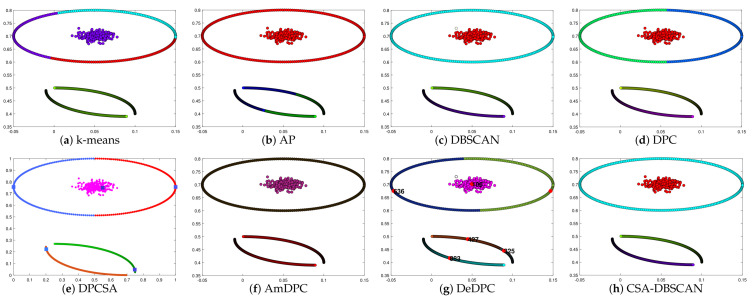
Clustering results on Donutcurves dataset.

**Figure 3:**
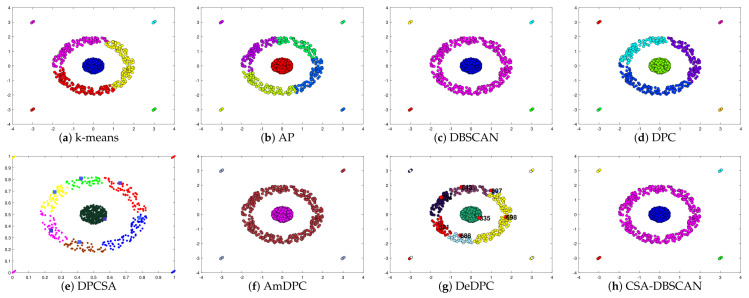
Clustering results on Target dataset.

**Figure 4:**
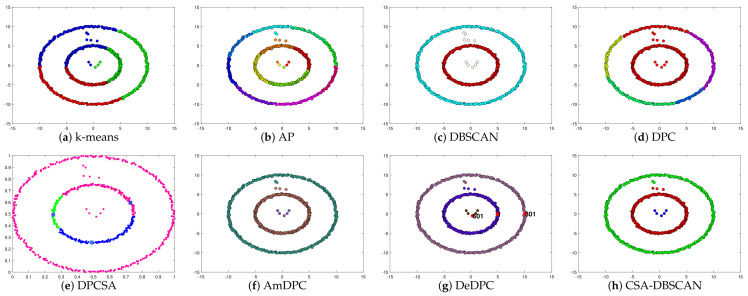
Clustering results on Twocirclesnoise dataset.

**Figure 5:**
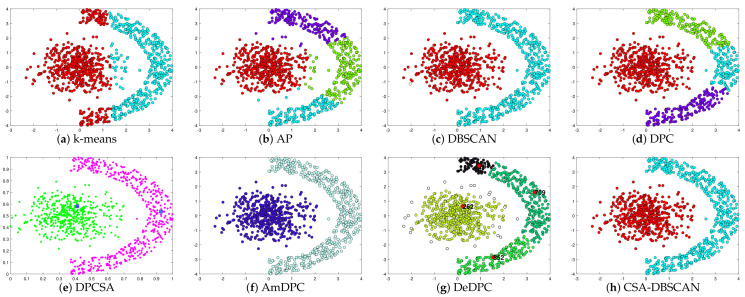
Clustering results on Pearl dataset.

**Figure 6:**
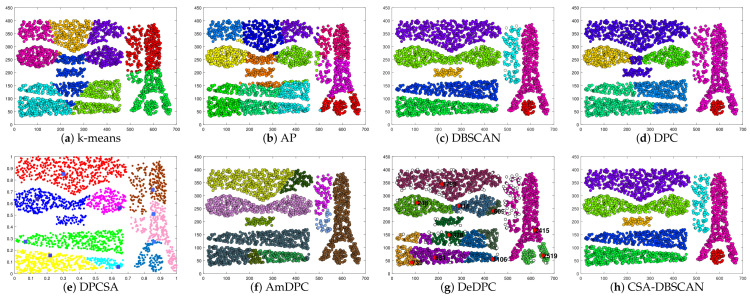
Clustering results on Complex8 dataset.

**Figure 7:**
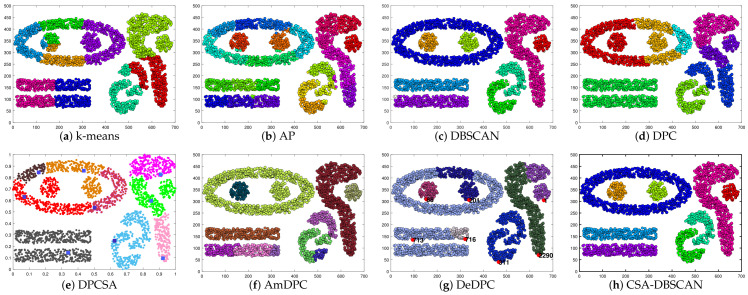
Clustering results on Complex9 dataset.

In the experiments, we kept the other parameters of the adaptive search constant and set the number of iterations to 100 to verify the adaptive effect of the improved method. The Twocirclesnoise and Target datasets for this experiment are datasets with anomalies as a separate class. Using this dataset allows further testing of the CSA-DBSCAN algorithm’s anomaly assignment performance. In Table 2, we give the evaluation metric values for the clustering of the eight state-of-the-art algorithms for the six synthetic datasets. The evaluation metric values for the different clustering methods correspond to the clustering results in Figure 2, Figure 3, Figure 4, Figure 5, Figure 6 and Figure 7. In this experiment, the best clustering results were obtained for all algorithms. Therefore, we analyzed the clustering accuracy and effectiveness of the algorithms in combination with the tabular and visual clustering results.

In Table 2, Par indicates the parameter settings of the method on different datasets. The visual clustering results of the method in Table 2 correspond to Figure 2, Figure 3, Figure 4, Figure 5, Figure 6 and Figure 7. In Table 2, the parameter of the k-means method is the quantity of clusters (*k*), the parameter of the AP method is the value of the matrix diagonal (*preference*), the parameters of the DBSCAN method are the quantities of neighborhoods (*MinPts*) and the domain radius (*Eps*), and the parameters of the DPC method are the average number of neighboring points, represented as a percentage (*p*). The DPCSA method is a parameterless clustering algorithm; the AmDPC model’s parameters are the number of neighbors ($N_{i}$), peak split lines ($C_{1}$, $C_{2}$) and average number of neighbors, represented as a percentage (*p*). The parameters of the DeDPC algorithm are the number of data point neighbors (*N*) and the average number of neighbors, represented as a percentage (*p*). The CSA-DBSCAN method can find the best *Eps* parameter adaptively, so the parameter of this method is *MinPts*. For the synthetic datasets, we uniformly set the search range of the CSA-DBSCAN method parameter to [0, 20]. Since the value of *MinPts* is a positive integer within 15, the CSA-DBSCAN algorithm nicely overcomes the drawback inherent to parameter setting in the DBSCAN approach.

In Table 2, we have marked the best clustering evaluation metric values for the different methods in bold. As can be seen in Table 2, the CSA-DBSCAN method obtained the best cluster evaluation metric values on the six synthetic datasets. Table 2 shows all the complex structured datasets with their uneven density distributions. It can be seen from the table that the k-means, AP, DPC, and DPCSA algorithms do not work well with this type of dataset, and AmDPC and DeDPC do not achieve the best metric values for some of the datasets, though the values they achieve are still high. The DBSCAN algorithm is very close to 1 for most of the clustering metric values but cannot reach 1. The main reason is that the algorithm incorrectly identifies the boundary points as anomalies in the clustering process. The outlier assignment mechanism of the CSA-DBSCAN algorithm solves the problem of incorrectly identified outliers in the DBSCAN method very well. The algorithm can adaptively find the best parameters and clustering data for any complex structure with good clustering accuracy and clustering performance.

To better analyze the clustering performance, in Figure 2, Figure 3, Figure 4, Figure 5, Figure 6 and Figure 7 and Table 2, all the algorithms correspond to the best clustering results. As shown in Figure 2, the Donutcurves dataset is a complex dataset of ring and semi-ring structures. We can see that the k-means, AP, DPC, DPCSA, AmDPC, and DeDPC methods do not accurately cluster the Donutcuvers dataset. The DBSCAN method clustered the data accurately but identified one of the category points as noisy, resulting in poor clustering accuracy. The CSA-DBSCAN approach obtained better clustering results in this complex structured dataset. In Figure 3, the Target dataset is a closed-loop dataset with an inhomogeneous density structure. We find that both the DBSCAN and CSA-DBSCAN algorithms can handle this type of dataset well. However, neither the AP nor the k-means algorithm can handle this type of dataset well. In Figure 4, we can see that both the k-means and AP algorithms do not handle the torus data and the central category points well. The DBSCAN approach uniformly misidentifies the central category points and the points outside the torus as noisy points. The CSA-DBSCAN method not only identifies the central category points but also assigns the points next to the torus to the corresponding clusters. It is possible to state that the CSA-DBSCAN method has good clustering accuracy and clustering efficiency for closed-loop datasets with irregular density compared to other algorithms.

In Figure 5, Pearl is a semi-annular multi-density dataset. We can see that the k-means, AP, DPC, and DeDPC algorithms are not able to handle the half-loop data, but both the DBSCAN and CSA-DBSCAN algorithms can handle this dataset well. However, the DBSCAN approach incorrectly identifies the boundary points as noisy points, while the DBSCAN, DPCSA, AmDPC, and CSA-DBSCAN algorithms obtain the best clustering results perfectly. In Figure 6, Complex8 is a dataset with a complex structure and an uneven density distribution, and the processing of this dataset can particularly detect the clustering effect of the method. In Figure 6, we can see that the k-means, AP, DPC, DPCSA, AmDPC, and DeDPC algorithms have poor clustering results and cannot identify complete classes for this dataset. It is encouraging that the CSA-DBSCAN method can obtain the optimal clustering effects more accurately than the DBSCAN algorithm. In Figure 7, the Complex9 dataset is a complex dataset with annular, semi-annular, and long arc structures. We can see that the k-means, AP, DPC, DPCSA, AmDPC, and DeDPC methods cannot handle this class of data. The DBSCAN and CSA-DBSCAN methods can handle this dataset perfectly, and the CSA-DBSCAN algorithm has better clustering accuracy and performance.

To further verify the utility of the proposed method, we further tested the effectiveness of the algorithm on real-world datasets.

### 4.3. Performance Evaluation on Real-World Datasets

In this subsection, we describe the clustering properties of different algorithms on real-world datasets. In Table 3, we give the values of clustering evaluation metrics for different methods on six high-dimensional datasets. The parameters of the algorithm tested on the real-world dataset are the same as those in Table 2. To facilitate comparison and analysis of the algorithm performance, we give in the table the values of the best clustering evaluation metrics obtained after repeated training and testing. Since real-world datasets have characteristics such as high dimensionality and nonlinearity, we uniformly set the search range of the CSA-DBSCAN method to [0, 20]. The search space of the proposed method is one parameter, so the search dimension is set to 1.

From Table 3, we can see that the CSA-DBSCAN algorithm for the Seeds dataset has higher values for all metrics than the other state-of-the-art clustering algorithms. From the Thyroid dataset, we can see that the NMI, ARI, and FM clustering metric values for the CSA-DBSCAN and DBSCAN methods are higher than the other algorithms, and the ACC metric values are slightly lower than the k-means method. In the Ionosphere dataset, the NMI, ARI, and FM clustering metrics of the CSA-DBSCAN algorithm are higher than the other algorithms, and the ACC metrics are slightly lower than those of the AmDPC method. In the Glass dataset, both the DeDPC and AmDPC algorithms have only one metric value higher than the other algorithms, respectively. In the Vehicle dataset, the ACC and FM metrics of the DeDPC algorithm are higher than the other algorithms, and the NMI and ARI metrics of the CSA-DBSCAN algorithm are higher than the other algorithms. In the Vehicle data set, the CSA-DBSCAN and DeDPC algorithms perform similarly. From the Iris dataset, we can see that the DPCSA algorithm achieves the best clustering evaluation metric values. The CSA-DBSCAN and AmDPC algorithms have the same and slightly lower clustering evaluation metric values than the DPCSA algorithm and are higher than the other clustering algorithms. Additionally, we can see that the CSA-DBSCAN algorithm only needs to input one parameter to obtain the best clustering result adaptively. Therefore, the parameters of the CSA-DBSCAN algorithm are simple and efficient. The CSA-DBSCAN method can redistribute these identified noisy points, which gives the new method better clustering capabilities and effectiveness in real-world datasets.

From Table 3, we can find that the clustering metric values of the DBSCAN algorithm differ from the clustering metric values of the CSA-DBSCAN algorithm. The main reason is that the data distribution in the high-dimensional dataset is discrete, and the DBSCAN method sometimes incorrectly identifies some boundary points as anomalies. The CSA-DBSCAN method can redistribute these identified noisy points, which makes the method better regarding clustering accuracy and effectiveness in the real dataset. In summary, the CSA-DBSCAN algorithm can handle datasets with a complex structure of inhomogeneous density.

The CSA-DBSCAN algorithm also has its unique advantages in practical applications. For complex image data, such as image segmentation and face recognition, the CSA-DBSCAN method can further explore the potential relationship between boundary points and anomalies, providing more reference values for image processing.

### 4.4. Performance Test of Outlier Processing after Fusion Deviation Theory

In this subsection, we will further explore the impact of introducing deviation theory to measure the compactness between data points on the clustering performance. We will select three representative synthetic datasets and two real-world datasets from Table 1 to fully measure the impact of deviation theory on the performance of outlier assignments. The nearest neighbor assignment mechanism of the CSA-DBSCAN algorithm is an extension of the KNN algorithm [33]. Before the use of deviation theory, the CSA-DBSCAN algorithm mainly relied on the KNN algorithm for the assignment of anomalies. Therefore, this assignment mechanism is influenced by the parameter *k*. Therefore, we will further discuss the effect of introducing the deviation theory and the variation of parameter value *k* on the clustering performance. We will control the other parameters of the CSA-DBSCAN algorithm in Table 2 and Table 3 to keep them constant and observe the effect of introducing deviation theory and different *k* values on the clustering results.

**Figure 8:**
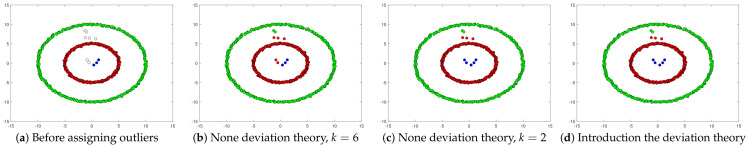
Results of outlier assignment on Twocirclesnoise dataset.

**Figure 9:**
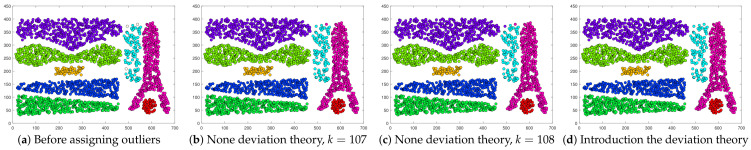
Results of outlier assignment on Complex8 dataset.

**Figure 10:**
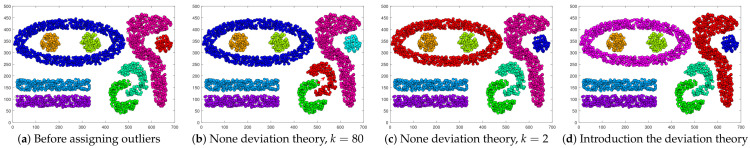
Results of outlier assignment on Complex9 dataset.

In Figure 8, Figure 9 and Figure 10, we present the results of the anomaly assignment for the three synthetic datasets Twocirclesnoise, Complex8, and Complex9. Figure 8a shows the results of the CSA-DBSCAN algorithm after iteration to obtain the optimal parameters, where the white points are the identified anomalies. Figure 8b,c shows the results of anomaly assignment for different parameter values *k* using the KNN method, respectively. After introducing the deviation theory to measure the compactness between data points, we directly assign the anomalies to the clustering results of the nearest category points according to the compactness relationship between the anomalies and other data points (see Figure 8d). Figure 9a and Figure 10a represent the anomaly identification results after iterations of the Complex8 and Complex9 datasets, respectively. In Figure 9b,c, we can see that some anomalies are assigned to other categories. In Figure 9d, we can see that the anomalies are well assigned to the corresponding categories. In Figure 10b, we can see that one outlier is incorrectly assigned to other classes along the direction of the red arrow. Both Figure 10c and Figure 10d display the accurate assignment of the outliers.

In Table 4, we give the values of the clustering evaluation metrics for the Twocirclesnoise, Complex8, Complex9, Seeds, and Vehicle datasets under different allocation strategies, respectively. Figure 8, Figure 9 and Figure 10 show the visual clustering results in Table 4. Seeds and Vehicle are real-world datasets. For easier observation, we bold the clustering results after introducing the deviation theory. From the table, we can see that different *k* values have an impact on the clustering performance, and the results of anomaly assignment using deviation theory have a significant improvement on the clustering performance. For the Seeds dataset, the clustering index value after introducing deviation theory is significantly higher than the KNN assignment method. In the Vehicle dataset, for NMI and ARI metric values, the method of assignation using the introduction of deviation theory always outperforms the method of assigning in other cases, and the variation in the values of ACC and FM is not significant. Therefore, overall, the Vehicle dataset has better performance for the assignment method with the introduction of deviation theory.

It can be seen that the KNN algorithm is used to directly assign outliers, and different parameter values *k* have a large impact on the clustering results. The deviation theory can measure the compactness between data points by processing the distance property in the data points. We assign anomalies directly to the nearest boundary points based on the compactness relationship between anomalies and category points. The assignment method with the introduction of deviation theory effectively solves the influence of the parameter *k* on the clustering performance and enables the CSA-DBSCAN algorithm to obtain more accurate clustering results. The assignment method with the introduction of deviation theory does not require any input parameters to complete the effective assignment of outliers. This allocation method greatly improves the effectiveness and stability of the CSA-DBSCAN algorithm’s outlier allocation.

### 4.5. Comparative Analysis of Algorithm Running Time

In this subsection, we compare and examine the algorithms’ running times in further detail. Before analyzing the algorithms’ running time, we provide the time complexity of the various methods (see Table 5). In Table 5, *n* represents the number of data points in the dataset, *I* represents the number of iterations, and *K* represents the number of clusters determined by the k-means method. According to Table 5, the DBSCAN algorithm has the lowest time complexity, followed by the k-means algorithm. The time complexity of the DPC, DPCSA, AmDPC, and DeDPC algorithms is similar and reasonable. Both the AP and CSA-DBSCAN algorithms are affected by the number of iterations during operation, so they have a higher time complexity. Theoretically, the time complexity of the AP algorithm is higher than that of the CSA-DBSCAN algorithm. However, the analysis of the runtime flow of the AP algorithm shows that the AP algorithm is only locally iterative during the runtime, while the CSA-DBSCAN algorithm is globally iterative during the runtime. In this case, it is difficult to further determine the magnitude of the time consumption of the AP and CSA-DBSCAN algorithms.

To further appreciate the time required to execute the various methods, we provide detailed running times for the 12 datasets in Table 2 and Table 3 (see Table 6). Each dataset in Table 6 corresponds to a different ordinal number. Table 6’s runtime parameter settings are identical to those in Table 2 and Table 3. As seen in Table 6, the DBSCAN algorithm has the shortest total runtime, while the k-means technique has the second shortest. The CSA-DBSCAN algorithm has the longest runtime compared to the other algorithms in the 12 datasets. Among them, the runtime consumption of the Complex8 and Complex9 datasets is 55.3762 and 86.5821 s, respectively, which far exceeds the runtime of the other algorithms. The Complex8 and Complex9 datasets have particularly large data volumes, and as a result, their runtime increases significantly. The CSA-DBSCAN algorithm is a fusion of CSA optimization and DBSCAN algorithm, and the overall running process is affected by the number of iterations, so the time the CSA-DBSCAN algorithm spends is higher than the other algorithms.

**Figure 11:**
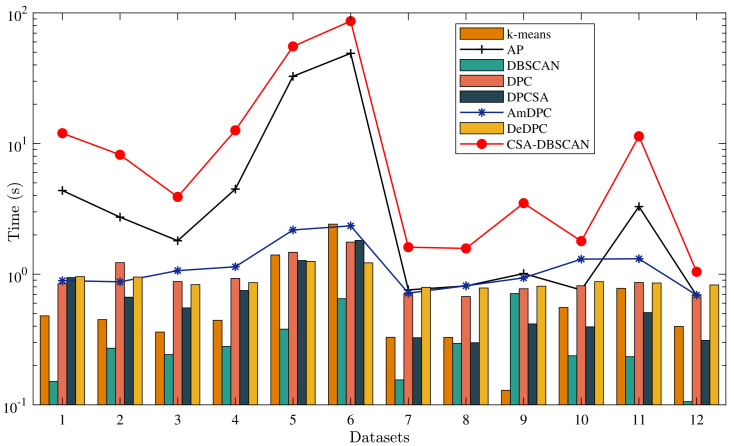
Comparison of the running time of different algorithms.

Table 6 includes a running time comparison (see Figure 11) to help visualize the time cost of the various algorithms. The CSA-DBSCAN, AP, and AmDPC algorithms have the longest overall runtimes, and runtimes greater than one second can be seen in Figure 11. We provide line comparison graphs of the CSA-DBSCAN, AP, and AmDPC algorithms to further observe the running time trends on different datasets (see Figure 11). In Figure 11, the horizontal coordinates represent the ordinal numbers corresponding to the different datasets in Table 6. We redesigned the scale of the vertical coordinates because of the excessive differences in the running times of the different algorithms. As a result, the time cost of different algorithms can all be compared and analyzed more intuitively. In Figure 11, we represent the algorithms with lower running times as bar charts. As can be seen in Figure 11, the runtime of the line graph is overall greater than one second, and the runtime of the bar graph is overall less than one second.

Figure 11 shows that the CSA-DBSCAN algorithm has the longest runtime, followed by the AP algorithm. The AmDPC algorithm has a shorter runtime than the CSA-DBSCAN and AP algorithms but a longer runtime than the others. The DBSCAN method has the shortest running time, followed by the k-means algorithm, while the DPC, DPCSA, and DeDPC algorithms have times that are comparable. The first six datasets in Figure 11 are synthetic, while the latter six are real-world datasets. From the description of the data attributes in Table 1, we can see that the amount of data in the synthetic dataset is significantly higher than that in the real-world dataset, and the running times of all eight algorithms are higher in the synthetic dataset than in the real-world dataset. In the real-world dataset, Ionosphere and Glass are both particularly high-dimensional datasets, but their runtime consumption is still lower than that of the synthetic dataset among these eight state-of-the-art algorithms.

Therefore, we may conclude that changing the dimensionality has little effect on the running time consumption of the algorithms. The amount of data in different datasets has a significant impact on an algorithm’s running time. Because the CSA-DBSCAN algorithm is a systematic fusion of CSA optimization and DBSCAN algorithm in an iterative process, its running time consumption is significant when compared to other algorithms.

### 4.6. Robustness Analysis of the CSA-DBSCAN Algorithm

In this subsection, we tested and analyzed the CSA-DBSCAN algorithm’s robustness. The CSA-DBSCAN algorithm is an adaptive method that fuses swarm intelligence optimization and a density clustering method. The method is divided into two stages: chameleon swarm optimization and density clustering. The process of optimizing the parameters of the DBSCAN algorithm using the chameleon swarm method is primarily influenced by two parameters: the number of iterations *I* and the number of populations *n*. We uniformly set these two parameters to fixed values of 60 and 10, respectively, before running the optimization algorithm. The DBSCAN algorithm is mainly influenced by the *MinPts* parameter. This parameter is usually set to a positive integer between 1 and 15. Therefore, we further verified the robustness of the CSA-DBSCAN method by analyzing the effect of different parameters on the clustering performance.

We separately tested the effects of the three CSA-DBSCAN algorithm parameters *I*, *n*, and *MinPts* on the clustering results and investigated their robustness. We began with the number of iterations *I*, which influences the CSA-DBSCAN algorithm’s parameter search quality. We set different values of *I* for testing: *I* ∈ [50, 500] (*I* is a positive integer), and then performed tests on three synthetic datasets and three real-world datasets, whose clustering results are shown in Table 7. The values in Table 7 are the means and standard deviations of the CSA-DBSCAN algorithm’s 10 experimental results for parameter *I* ranging from 50 to 500. We highlighted the mean and standard deviation of the better evaluation metrics’ results. To ensure experiment reliability, we kept the population size parameters *n* and *MinPts* constant to test the effect of the CSA-DBSCAN algorithm parameter *I* on clustering performance.

As expressed in Table 7, the mean values of the assessment metrics for the datasets Twocirclesnoise, Pearl, Complex8, and Glass are greater and have a standard deviation of 0, which suggests that the number of iterations does not affect the clustering outcomes. The mean values and low standard deviations of the evaluation measures for the Vehicle and Iris datasets are comparable to those in Table 3. Therefore, the clustering performance of the CSA-DBSCAN algorithm is sound for the Vehicle and Iris datasets. Generally, the variation of the clustering performance of the CSA-DBSCAN algorithm with the number of iterations *I* is robust. The main reason for the variation in the clustering results for the Vehicle and Iris datasets is that the optimization process is not optimal at 50 iterations. In the experimental process, we only need to set the number of iterations to be greater than 50 to achieve the optimal optimization of the algorithm.

In Table 8, we give a range of values for the number of chameleon populations, *n*. For the experiments, we set *n* to be between 5 and 55 and divided it into ten equal parts. Table 8 shows the mean and standard deviation of the clustering index values for ten experiments with the number of iterations *I* and *MinPts* held constant. The better values are highlighted in bold. From Table 8, we can see that the mean values of the indicators for the six datasets are the same as those in Table 2 and Table 3 and have zero standard deviation. Thus, it can be seen that the changes in the number of chameleon populations *n* and the number of iterations *I* hardly affect the clustering results. Therefore, the number of iterations *I* and the number of chameleon populations *n* are robust in the clustering process.

As shown in Table 9, we maintained the number of iterations *I* and the number of populations *n* of the optimization process as constant values to see how the DBSCAN algorithm parameter *MinPts* affects the clustering results. We set *MinPts* from 1 to 10 and split them into 10 equal portions. By computing the mean and standard deviation of the clustering metrics for the six datasets at 10 different *MinPts*, we were able to determine the sensitivity of the DBSCAN and CSA-DBSCAN algorithms to parameter changes. For easy observation, we bolded the improved mean and standard deviation in Table 9. From Table 9, we can see that the mean values of the CSA-DBSCAN algorithm are always higher than the DBSCAN algorithm for the six datasets. For the Twocirclesnoise, Pearl, and Complex8 datasets, the standard deviations of the CSA-DBSCAN algorithm are always lower than those of the DBSCAN method. This indicates that the CSA-DBSCAN algorithm is more robust with the variation of *MinPts* in these three datasets. The standard deviations of a few measures in the DBSCAN algorithm are marginally lower than those in the CSA-DBSCAN algorithm in the Glass, Vehicle, and Iris datasets but are otherwise extremely close. This indicates that the clustering results of the CSA-DBSCAN algorithm are better and more robust in these six datasets. This shows that when the *MinPts* values are changed, the CSA-DBSCAN algorithm is more robust than the DBSCAN method. In summary, the CSA-DBSCAN algorithm considerably enhances the DBSCAN algorithm’s soundness.

To examine the significance of the differences in performance between the CSA-DBSCAN and DBSCAN algorithms with different*MinPts* settings in Table 9, we conduct statistical tests, as shown in Table 10. In Table 10, we use *p*-values to determine if there is a statistical difference in algorithm performance based on NMl, ARI, ACC, and FM evaluation criteria. As shown in Table 10, the *p*-values for the Pearl, Glass, and Iris datasets are less than 0.05, and in some cases, less than 0.01 for most measures, demonstrating that a performance difference between the CSA-DBSCAN and DBSCAN algorithms exists and is extremely statistically significant. Only the *p*-value for the NMI metric in the Twocirclesnoise and Complex8 datasets was less than 0.05, indicating a statistically significant difference between the NMI values in these two datasets. In the Vehicle dataset, the *p*-values for the ARI and FM measures are less than 0.01, showing a highly significant difference between these two metrics. In Table 10, we highlight the values of the indicators with significant differences. Combining the mean and standard deviation of the evaluated metric values in Table 9, we can see that the CSA-DBSCAN algorithm outperforms the DBSCAN method in general. Therefore, for the *MinPts* parameter, the CSA-DBSCAN algorithm is overall more robust than DBSCAN and has a statistically significant difference.

In conclusion, as the number of iterations *I*, the number of populations *n*, and the value of *MinPts* are modified, the CSA-DBSCAN algorithm can still produce superior clustering results. The clustering performance of the CSA-DBSCAN algorithm has a certain robustness and effectiveness.

### 4.7. Color Image Segmentation Clustering Applications

**Figure 12:**
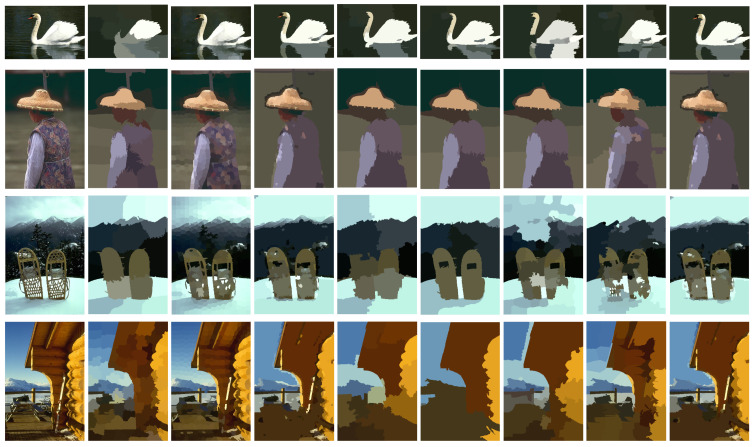
Image segmentation results of different clustering algorithms.

In this subsection, we selected four real images from the Berkeley segmentation dataset (BSDS500) to test the clustering performance of the CSA-DBSCAN algorithm’s image segmentation. In this experiment, the image segmentation evaluation metric $Fit_{BDE}$ was chosen as the fitness function to adaptively search for the optimal parameter values. In Figure 12, the original image is shown on the leftmost side, and the image segmentation results of eight state-of-the-art clustering methods, k-means, AP, DBSCAN, DPC, DPCSA, AmDPC, DeDPC, and CSA-DBSCAN, are shown from left to right. In order to reduce the computational effort, an effective superpixel SLIC algorithm was used in this paper to pre-segment the image into multiple superpixel information uniformly before image processing. Finally, this information is fed into the different algorithms mentioned above for testing.

To evaluate the effectiveness of the CSA-DBSCAN algorithm for practical applications in image segmentation, we give the boundary displacement error (BDE) [32] and probabilistic Rand index (PRI) metrics [38] for different image segmentation methods. PRI is calculated as the ratio of the number of pixels with identical algorithmic segmentation and multiple manual segmentation labels to the whole number of pixels; BDE is a measure of the average displacement error of the boundary pixels in both segmentation results. In Table 11, we give the segmentation metric metrics for the four real images in Figure 12 in order from top to bottom. The “↓” indicates that the smaller the BDE value is, the better the image segmentation is. The “↑” indicates that the larger the PRI value, the better the image segmentation effect, and the PRI value is in the range of (0, 1). The evaluation index values in Table 11 and Table 12 correspond to the clustering results in Figure 12. In the table, we bolded the best indicator values for a clearer view of the data.

From Table 11 and Table 12 and Figure 12, we can see that the first three image k-means, AP, DPC, DPCSA, AmDPC, and DeDPC algorithms counting from top to bottom do not segment the images as well as the DBSCAN and CSA-DBSCAN algorithms. Both the DBSCAN and CSA-DBSCAN methods segment the images well and accurately describe the overall image contour. In the last image, the AmDPC algorithm PRI metric value reaches its highest value, and the CSA-DBSCAN algorithm PRI value is very close compared to AmDPC. However, the BDE metric value of the CSA-DBSCAN algorithm is much higher than that of the AmDPC algorithm. Overall, the CSA-DBSCAN algorithm outperforms the AmDPC algorithm for the segmentation of the fourth image. As can be seen in Table 11 and Table 12, the BDE and PRI metric values of the DBSCAN method are slightly lower than those of the CSA-DBSCAN method. This indicates that the CSA-DBSCAN method has better image segmentation accuracy compared to the DBSCN algorithm.

Therefore, compared with other mainstream clustering algorithms, the CSA-DBSCAN algorithm has better clustering accuracy and effectiveness in the application of image segmentation. In summary, the CSA-DBSCAN algorithm has a certain effectiveness and practicality in adaptive search parameters and image segmentation.

## 5. Discussion

This research focuses on the fusion strategy of the swarm intelligence optimization adaptive clustering algorithm. Firstly, the *Eps* parameters of DBSCAN are utilized as individual candidate solutions for chameleons, and the optimal *Eps* clustering parameter values are obtained adaptively by the optimization mechanism of the CSA algorithm; then, the identified anomalies are found. Finally, the nearest neighbor mechanism of deviation theory is used to reacquire the compactness relationship between the data and assign the anomalies to the proximity cluster classes to obtain the final clustering results. The CSA-DBSCAN algorithm takes advantage of the CSA optimization and DBSCAN clustering performance to form a new adaptive clustering fusion algorithm. Compared with other algorithms, the CSA-DBSCAN algorithm can adaptively obtain the best clustering parameter values and clustering results, reducing the complexity of clustering tuning, enabling the clustering of arbitrarily structured complex datasets and the identification and assignment of outliers.

To boost computational efficiency in image processing, the CSA-DBSCAN algorithm employs the SLIC method to create superpixel information on the original image. The method then uses an updated way to segment the superpixel data based on the image color space features to achieve image segmentation using the adaptive CSA-DBSCAN method. The approach of pre-processing color photos with SLIC significantly reduces the algorithm’s computing time consumption. The CSA-DBSCAN algorithm is a single-parameter adaptive clustering algorithm. To discover the optimum clustering result quickly and reliably, the technique requires only one integer number parameter. When compared to existing algorithms, the modified approach performs exceptionally well in adaptive search and image segmentation.

The CSA-DBSCAN algorithm can handle color images well, but its performance for vector image data in space is unknown. For vector image data clustering, the DBSCAN algorithm’s Euclidean distance calculation is insufficient to tackle the associated special challenges. In the subsequent study, we will use the spatial clustering method with network path distances proposed by Yu [39], combined with the advantages of the CSA-DBSCAN algorithm, to cluster vector image data through a preprocessing step of mathematical morphology and vector-to-raster conversion.

## 6. Conclusions

In this research, we employ the most recent chameleon swarm optimization method for the adaptive optimization of the DBSCAN clustering algorithm parameter *Eps*, which solves the problem of *Eps* being particularly sensitive to clustering outcomes. To obtain the optimal clustering outcome, the CSA-DBSCAN algorithm requires only one parameter to be entered during operation. We employ the closest neighbor mechanism and deviation theory to assign noisy points to the nearest boundary points in the DBSCAN method to solve the problem of the over-identification of noisy points. The simulation results show that the improved CSA-DBSCAN method can handle complex structural data with arbitrary irregular density and has higher clustering accuracy and effect in high-dimensional datasets. The CSA-DBSCAN algorithm implements the fusion of the swarm intelligence algorithm, clustering algorithm, nearest neighbor mechanism, and deviation theory. Compared with other algorithms, this algorithm can find the best clustering results quickly and identify outliers more accurately, with better clustering precision and performance. The image processing experiment results suggest that the CSA-DBSCAN algorithm has a high practical value in the field of image segmentation.

However, the CSA-DBSCAN algorithm must also change the *MinPts* parameter, which requires considerable tuning time for unknown data. We will apply multi-objective optimization algorithms with stronger optimization capabilities in the future to achieve optimal clustering with the benefit of parameter-free clustering.

## Figures and Tables

**Table 1:** Description of the experimental datasets.

Dataset	Objects	Dimension	Clusters
Donutcurves	1000	2	4
Target	770	2	6
Twocirclesnoise	610	2	3
Pearl	1000	2	2
Complex8	2551	2	8
Complex9	3031	2	9
Seeds	210	7	3
Thyroid	215	5	3
Ionosphere	351	30	2
Glass	214	9	6
Vehicle	846	18	4
Iris	150	4	3

**Table 2:** Description of clustering evaluation on synthetic datasets.

Datasets	Algorithms	Par	NMI	ARI	ACC	FM
Donutcurves	k-means	4	0.6821	0.1525	0.5840	0.6796
	AP	−6.4125	0.5802	0.4085	0.5290	0.6139
	DBSCAN	0.01/2	0.9977	0.9987	0.9990	0.9990
	DPC	3	0.9429	0.9130	0.8810	0.9353
	DPCSA	-	0.9428	0.9128	0.8750	0.9351
	AmDPC	3/0.05/0.15/8	0.8660	0.5551	0.7500	0.8160
	DeDPC	11/5	0.8966	0.8240	0.7820	0.8702
	**CSA-DBSCAN**	**2**	**1.0000**	**1.0000**	**1.0000**	**1.0000**
Target	k-means	6	0.7507	0.1048	0.7000	0.8306
	AP	−82.8259	0.6905	0.6557	0.6532	0.8055
	**DBSCAN**	**0.3/2**	**1.0000**	**1.0000**	**1.0000**	**1.0000**
	DPC	20	0.7767	0.7020	0.7052	0.8341
	DPCSA	-	0.6525	0.6234	0.6351	0.7850
	AmDPC	5/1/0.39/0.3	0.9771	0.9924	0.9883	0.9961
	DeDPC	8/2	0.6698	0.6519	0.7078	0.8031
	**CSA-DBSCAN**	**2**	**1.0000**	**1.0000**	**1.0000**	**1.0000**
Twocirclesnoise	k-means	3	0.0039	-0.2200	0.3377	0.4026
	AP	−97.4305	0.5205	0.1860	0.2197	0.4281
	DBSCAN	0.8/4	0.9574	0.9835	0.9918	0.9916
	DPC	8	0.4396	0.3814	0.6262	0.6619
	DPCSA	-	0.3574	0.2663	0.6951	0.6456
	**AmDPC**	**3/1.6/1.9/5**	**1.0000**	**1.0000**	**1.0000**	**1.0000**
	**DeDPC**	**1/2**	**1.0000**	**1.0000**	**1.0000**	**1.0000**
	**CSA-DBSCAN**	**3**	**1.0000**	**1.0000**	**1.0000**	**1.0000**
Pearl	k-means	2	0.3920	0.4511	0.8360	0.7310
	AP	−158.3515	0.6454	0.6067	0.6620	0.7790
	DBSCAN	0.575/4	0.9948	0.9980	0.9990	0.9990
	DPC	8	0.7848	0.6682	0.6860	0.8173
	**DPCSA**	**-**	**1.0000**	**1.0000**	**1.0000**	**1.0000**
	**AmDPC**	**3/2/2/5**	**1.0000**	**1.0000**	**1.0000**	**1.0000**
	DeDPC	17/2	0.7556	0.6933	0.7480	0.8325
	**CSA-DBSCAN**	**4**	**1.0000**	**1.0000**	**1.0000**	**1.0000**
Complex8	k-means	8	0.6129	−0.0430	0.5214	0.4954
	AP	−6349.1939	0.6440	0.3936	0.4606	0.4891
	DBSCAN	15.58/3	0.9985	0.9996	0.9988	0.9997
	DPC	5	0.7812	0.5113	0.7327	0.7177
	DPCSA	-	0.8405	0.7470	0.7589	0.7907
	AmDPC	3/50/38.6/16	0.8574	0.7528	0.8009	0.7955
	DeDPC	20/2	0.7458	0.6264	0.6452	0.6876
	**CSA-DBSCAN**	**2**	**1.0000**	**1.0000**	**1.0000**	**1.0000**
Complex9	k-means	9	0.6684	0.0270	0.4985	0.3145
	AP	−2972.5168	0.7380	0.2845	0.3583	0.4393
	DBSCAN	15.12/10	0.9942	0.9975	0.9970	0.9979
	DPC	3	0.7167	0.4462	0.5737	0.5627
	DPCSA	-	0.7199	0.4221	0.4593	0.5188
	AmDPC	3/20/21/50	0.9132	0.8919	0.8746	0.9132
	DeDPC	1/2	0.7297	0.3389	0.6457	0.6213
	**CSA-DBSCAN**	**8**	**1.0000**	**1.0000**	**1.0000**	**1.0000**

**Table 3:** Description of clustering evaluation on real-world datasets.

Datasets	Algorithms	Par	NMI	ARI	ACC	FM
Seeds	k-means	3	0.6949	0.7166	0.8952	0.8106
	AP	−61.1525	0.7101	0.7103	0.8905	0.8068
	DBSCAN	0.92/4	0.4823	0.4001	0.5952	0.6439
	DPC	6	0.7188	0.7132	0.8905	0.8093
	DPCSA	-	0.7151	0.7236	0.8952	0.8166
	AmDPC	10/1.5/3/10	0.7188	0.7132	0.8905	0.8093
	DeDPC	15/15	0.7188	0.7132	0.8905	0.8093
	**CSA-DBSCAN**	**11**	**0.7259**	**0.7321**	**0.9000**	**0.8212**
Thyroid	**k-means**	**3**	0.4354	0.5823	**0.8558**	0.8062
	AP	−15.0333	0.4728	0.0845	0.1535	0.3027
	**DBSCAN**	**4.28/1**	**0.5013**	**0.6915**	0.7209	**0.8648**
	DPC	6	0.3764	0.3535	0.7860	0.7828
	DPCSA	-	0.3517	0.3185	0.7954	0.7750
	AmDPC	15/5.8/5/2	0.4256	0.4114	0.8233	0.7952
	DeDPC	10/15	0.4313	0.4240	0.8279	0.7978
	**CSA-DBSCAN**	**1**	**0.5013**	**0.6915**	0.7209	**0.8648**
Ionosphere	k-means	2	0.1292	0.1681	0.7066	0.6004
	AP	−80.4075	0.1243	0.1634	0.7037	0.5983
	DBSCAN	1.4/1	0.3697	0.5112	0.6068	0.7526
	DPC	2	0.2669	0.2518	0.6581	0.6082
	DPCSA	-	0.1224	0.1969	0.7265	0.6335
	**AmDPC**	**3/1.3/0.87/2**	0.3212	0.3927	**0.8177**	0.7553
	DeDPC	3/5	0.2586	0.2612	0.7123	0.6453
	**CSA-DBSCAN**	**1**	0.6154	**0.3736**	**0.5289**	**0.7640**
Glass	k-means	6	0.4353	0.2389	0.4766	0.5493
	AP	−46.6739	0.2969	0.1750	0.4439	0.5408
	DBSCAN	0.5/4	0.3327	0.1771	0.4159	0.4381
	DPC	1	0.3053	0.1707	0.4579	0.4784
	DPCSA	-	0.2971	0.1772	0.4533	0.5384
	**AmDPC**	**4/1.5/3/5**	0.4008	0.1946	0.4860	**0.5700**
	**DeDPC**	**18/28**	0.4540	0.2510	**0.5093**	0.4938
	**CSA-DBSCAN**	**1**	**0.5269**	**0.2930**	0.4673	0.5570
Vehicle	k-means	4	0.1163	0.0783	0.3499	0.3422
	AP	−30.7022	0.1876	0.1165	0.3440	0.3085
	DBSCAN	0.55/10	0.2146	0.0602	0.4031	0.3993
	DPC	2	0.1603	0.0864	0.3723	0.4280
	**DPCSA**	**-**	0.2021	0.1098	**0.4054**	0.4074
	AmDPC	5/0.77/0.71/0.8	0.1863	0.0116	0.3759	0.4333
	**DeDPC**	**17/5**	0.1953	0.0028	**0.4054**	**0.4349**
	**CSA-DBSCAN**	**4**	**0.2160**	**0.1266**	0.3410	0.3569
Iris	k-means	3	0.7582	0.8933	0.7302	0.8208
	AP	−34.9374	0.7612	0.6667	0.3705	0.7715
	DBSCAN	0.4/5	0.7196	0.7063	0.8067	0.7972
	DPC	2	0.8058	0.7592	0.9067	0.8407
	**DPCSA**	**-**	**0.8851**	**0.9038**	**0.9667**	**0.9355**
	AmDPC	4/1/1/1	**0.8705**	**0.8858**	**0.9600**	**0.9234**
	DeDPC	5/2	0.8058	0.7592	0.9067	0.8407
	**CSA-DBSCAN**	**5**	**0.8705**	**0.8858**	**0.9600**	**0.9234**

**Table 4:** Clustering evaluation index values of different outlier allocation strategies.

Datasets	Allocation Strategy	NMI	ARI	ACC	FM
Twocirclesniose	*k* = 6	0.9827	0.9934	0.9967	0.9967
	*k* = 2	1.0000	1.0000	1.0000	1.0000
	Deviation theory	**1.0000**	**1.0000**	**1.0000**	**1.0000**
Complex8	*k* = 107	0.9986	0.9994	0.9996	0.9995
	*k* = 108	0.9973	0.9987	0.9992	0.9989
	Deviation theory	**1.0000**	**1.0000**	**1.0000**	**1.0000**
Complex9	*k* = 80	0.9989	0.9997	0.9997	0.9998
	*k* = 2	1.0000	1.0000	1.0000	1.0000
	Deviation theory	**1.0000**	**1.0000**	**1.0000**	**1.0000**
Seeds	*k* = 3	0.7101	0.7103	0.8905	0.8068
	*k* = 4	0.7114	0.7298	0.9000	0.8195
	Deviation theory	**0.7259**	**0.7321**	**0.9000**	**0.8212**
Vehicle	*k* = 4	0.2149	0.1159	0.3428	0.3595
	*k* = 14	0.1855	0.0970	0.3475	0.3649
	Deviation theory	**0.2160**	**0.1266**	**0.3416**	**0.3569**

**Table 5:** Time complexity comparison.

Algorithms	k-Means	AP	DBSCAN	DPC	DPCSA	AmDPC	DeDPC	CSA-DBSCAN
Time Complexity	O(IKn)	$O(In^{2})$	O(nlogn)	$O(n^{2})$	$O(n^{2})$	$O(n^{2})$	$O(n^{2})$	O(Inlogn)

**Table 6:** Running time for different algorithms (in *s*).

No.	Datasets	k-Means	AP	DBSCAN	DPC	DPCSA	AmDPC	DeDPC	CSA-DBSCAN
1	Donutcurves	0.4799	4.3714	0.1511	0.8441	0.9428	0.8927	0.9582	**11.9972**
2	Target	0.4493	2.7322	0.2713	1.2256	0.6670	0.8741	0.9513	**8.2078**
3	Twocirclesnoise	0.3607	1.8038	0.2427	0.8785	0.5524	1.0653	0.8324	**3.9055**
4	Pearl	0.4438	4.4842	0.2799	0.9278	0.7504	1.1395	0.8617	**12.6280**
5	Complex8	1.4032	32.7125	0.3798	1.4718	1.2732	2.1808	1.2533	**55.3762**
6	Complex9	2.4172	49.0449	0.6496	1.7611	1.8137	2.3449	1.2213	**86.5821**
7	Seeds	0.3289	0.7586	0.1553	0.7071	0.3264	0.7168	0.7937	**1.6091**
8	Thyroid	0.3285	0.8131	0.2953	0.6746	0.2989	0.8153	0.7843	**1.5749**
9	Ionosphere	0.1292	1.0116	0.7109	0.7737	0.4164	0.9375	0.8096	**3.4974**
10	Glass	0.5561	0.7568	0.2375	0.8159	0.3957	1.3038	0.8764	**1.7904**
11	Vehicle	0.7794	3.2929	0.2337	0.8635	0.5084	1.3128	0.8567	**11.3781**
12	Iris	0.3983	0.6863	0.1059	0.6982	0.3115	0.6915	0.8273	**1.0434**

**Table 7:** Robustness analysis of the CSA-DBSCAN algorithm with different numbers of iterations *I*.

Datasets *I* ∈ [50, 500]	NMI	ARI	ACC	FM
Twocirclesnoise	**1.0000** ± **0.0000**	**1.0000** ± **0.0000**	**1.0000** ± **0.0000**	**1.0000** ± **0.0000**
Pearl	**1.0000** ± **0.0000**	**1.0000** ± **0.0000**	**1.0000** ± **0.0000**	**1.0000** ± **0.0000**
Complex8	**1.0000** ± **0.0000**	**1.0000** ± **0.0000**	**1.0000** ± **0.0000**	**1.0000** ± **0.0000**
Glass	**0.5269** ± **0.0000**	**0.2930** ± **0.0000**	**0.4673** ± **0.0000**	**0.5570** ± **0.0000**
Vehicle	**0.1957** ± **0.0584**	**0.1133** ± **0.0380**	**0.3337** ± **0.0246**	**0.3722** ± **0.0435**
Iris	**0.8486** ± **0.0461**	**0.7827** ± **0.2173**	**0.9013** ± **0.1237**	**0.8930** ± **0.0641**

**Table 8:** Robustness analysis of the CSA-DBSCAN algorithm with different chameleon numbers *n*.

Datasets *n* ∈ [5, 55]	NMI	ARI	ACC	FM
Twocirclesnoise	**1.0000** ± **0.0000**	**1.0000** ± **0.0000**	**1.0000** ± **0.0000**	**1.0000** ± **0.0000**
Pearl	**1.0000** ± **0.0000**	**1.0000** ± **0.0000**	**1.0000** ± **0.0000**	**1.0000** ± **0.0000**
Complex8	**1.0000** ± **0.0000**	**1.0000** ± **0.0000**	**1.0000** ± **0.0000**	**1.0000** ± **0.0000**
Glass	**0.5269** ± **0.0000**	**0.2930** ± **0.0000**	**0.4673** ± **0.0000**	**0.5570** ± **0.0000**
Vehicle	**0.2160** ± **0.0000**	**0.1266** ± **0.0000**	**0.3416** ± **0.0000**	**0.3569** ± **0.0000**
Iris	**0.8705** ± **0.0000**	**0.8858** ± **0.0000**	**0.9600** ± **0.0000**	**0.9234** ± **0.0000**

**Table 9:** Robustness analysis of the CSA-DBSCAN algorithm with different values of *MinPts*.

Datasets *MinPts* ∈ [1, 10]	Algorithms	NMI	ARI	ACC	FM
Twocirclesniose	DBSCAN	0.8815 ± 0.1558	0.9005 ± 0.1716	0.9234 ± 0.1453	0.9434 ± 0.0997
	CSA-DBSCAN	**0.9827** ± **0.0149**	**0.9902** ± **0.0085**	**0.9951** ± **0.0042**	**0.9951** ± **0.0042**
Pearl	DBSCAN	0.9903 ± 0.0076	0.9941 ± 0.0028	0.9970 ± 0.0014	0.9970 ± 0.0014
	CSA-DBSCAN	**0.9990** ± **0.0032**	**0.9996** ± **0.0013**	**0.9998** ± **0.0006**	**0.9998** ± **0.0006**
Complex8	DBSCAN	0.9644 ± 0.0488	0.9620 ± 0.0635	0.9545 ± 0.0656	0.9691 ± 0.0514
	CSA-DBSCAN	**0.9840** ± **0.0181**	**0.9876** ± **0.0172**	**0.9784** ± **0.0242**	**0.9898** ± **0.0142**
Glass	DBSCAN	0.2902 ± 0.1138	0.1194 ± 0.0802	0.3925 ± **0.0336**	0.4231 ± 0.0193
	CSA-DBSCAN	**0.3580** ± **0.0885**	**0.2248** ± **0.0447**	**0.4579**± 0.0337	**0.5474** ± **0.0111**
Vehicle	DBSCAN	0.1658 ± **0.0407**	0.0561 ± 0.0375	0.3369 ± 0.0563	0.3529 ± **0.0220**
	CSA-DBSCAN	**0.1878**± 0.0481	**0.1022** ± **0.0202**	**0.3482** ± **0.0175**	**0.3724**± 0.0252
Iris	DBSCAN	0.6710 ± 0.1003	0.5889 ± **0.1051**	0.7427 ± **0.0563**	0.7198 ± 0.0682
	CSA-DBSCAN	**0.8023** ± **0.0450**	**0.7219**± 0.1921	**0.8493**± 0.1054	**0.8511** ± **0.0547**

**Table 10:** The robustness statistics test results (*p*-value) with different *MinPts* values.

Datasets *MinPts* ∈ [1, 10]	NMI	ARI	ACC	FM
Twocirclesnoise	**0.0309**	0.0630	0.0768	0.0658
Pearl	**0.0002**	**0.0002**	**0.0003**	**0.0002**
Complex8	**0.0437**	0.0640	0.0634	0.0626
Glass	**0.0001**	**0.0000**	**0.0000**	**0.0000**
Vehicle	0.1569	**0.0078**	0.2147	**0.0031**
Iris	**0.0000**	**0.0148**	**0.0091**	**0.0000**

**Table 11:** Image segmentation BDE**↓** index comparison.

No.	k-Means	AP	DBSCAN	DPC	DPCSA	AmDPC	DeDPC	CSA-DBSCAN
Image 1	15.7662	19.7150	5.0937	15.4732	8.5149	7.4530	10.7306	**4.9178**
Image 2	11.5680	14.2134	7.6185	9.9451	9.9451	11.2650	13.5390	**7.5544**
Image 3	9.5631	12.2107	6.7734	10.0532	7.1654	11.9157	9.5474	**6.1981**
Image 4	16.4136	16.5830	12.5754	18.8168	11.8731	12.0519	13.6472	**11.7218**

**Table 12:** Image segmentation PRI**↑** index comparison.

No.	k-Means	AP	DBSCAN	DPC	DPCSA	AmDPC	DeDPC	CSA-DBSCAN
Image 1	0.6063	0.5410	0.8875	0.6402	0.6851	0.6757	0.6227	**0.8927**
Image 2	0.8467	0.8205	0.9047	0.8814	0.8814	0.8657	0.8323	**0.9051**
Image 3	0.8551	0.8146	0.8934	0.8593	0.8945	0.8252	0.5606	**0.8997**
Image 4	0.7479	0.7403	0.7887	0.7730	0.7823	**0.8083**	0.7615	**0.7990**

## Data Availability

The real-world datasets can be obtained from https://archive.ics.uci.edu/ml/index.php (accessed on on 14 July 2022). The BSD500 datasets can be obtained from https://www2.eecs.berkeley.edu/Research/Projects/CS/vision/grouping/resources.html (accessed on 25 June 2022).
